# Supplementary figures and images for: Simulation framework for generating intratumor heterogeneity patterns in a cancer cell population
Source: PLoS One. 2017 Sep 6;12(9):e0184229. doi: 10.1371/journal.pone.0184229 (PMC5587296; doi:10.1371/journal.pone.0184229)

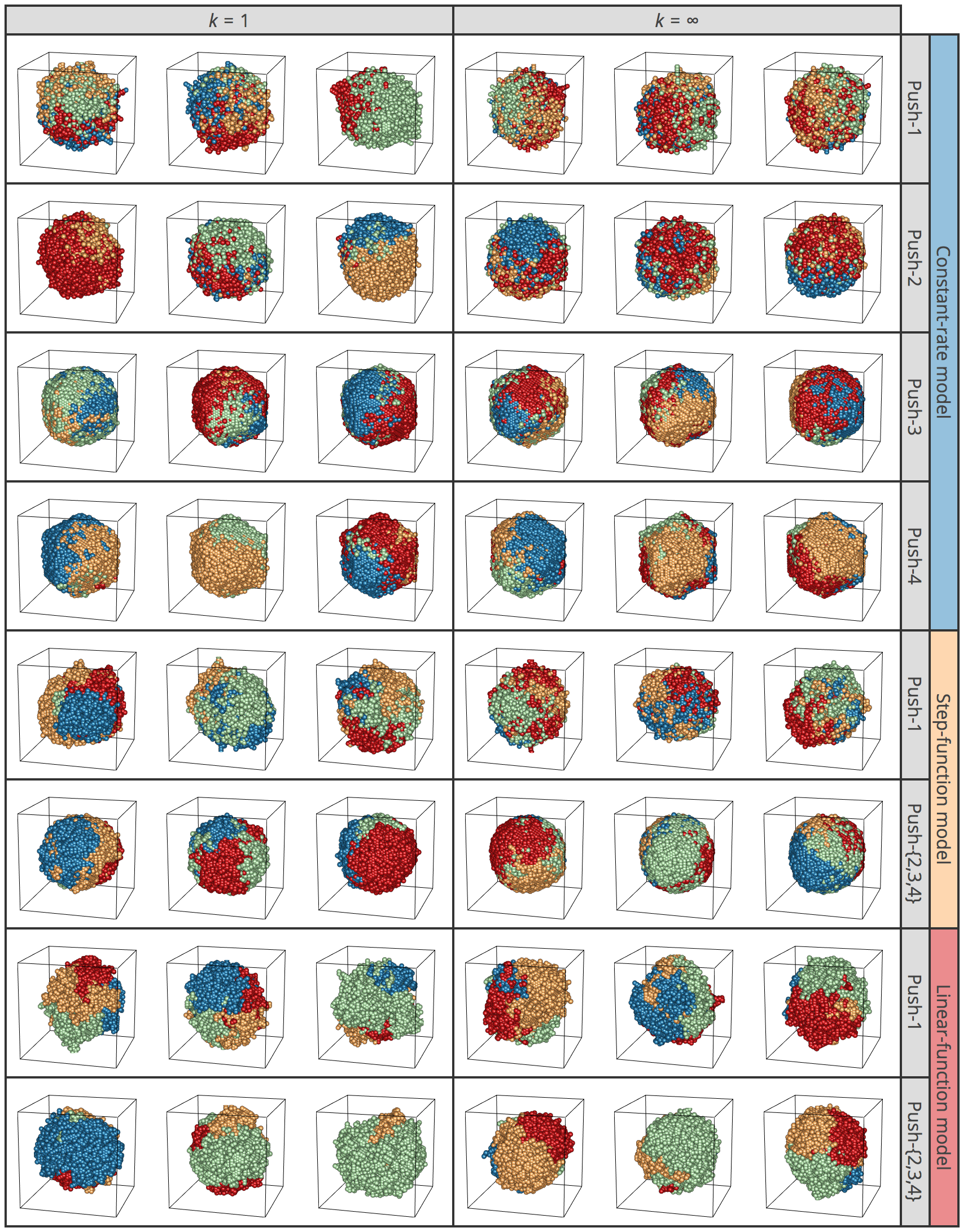

Supplement: S1 Fig — All parameters except for the lattice/neighborhood are the same as those in Fig 8. (TIF) [file pone.0184229.s001.tif]

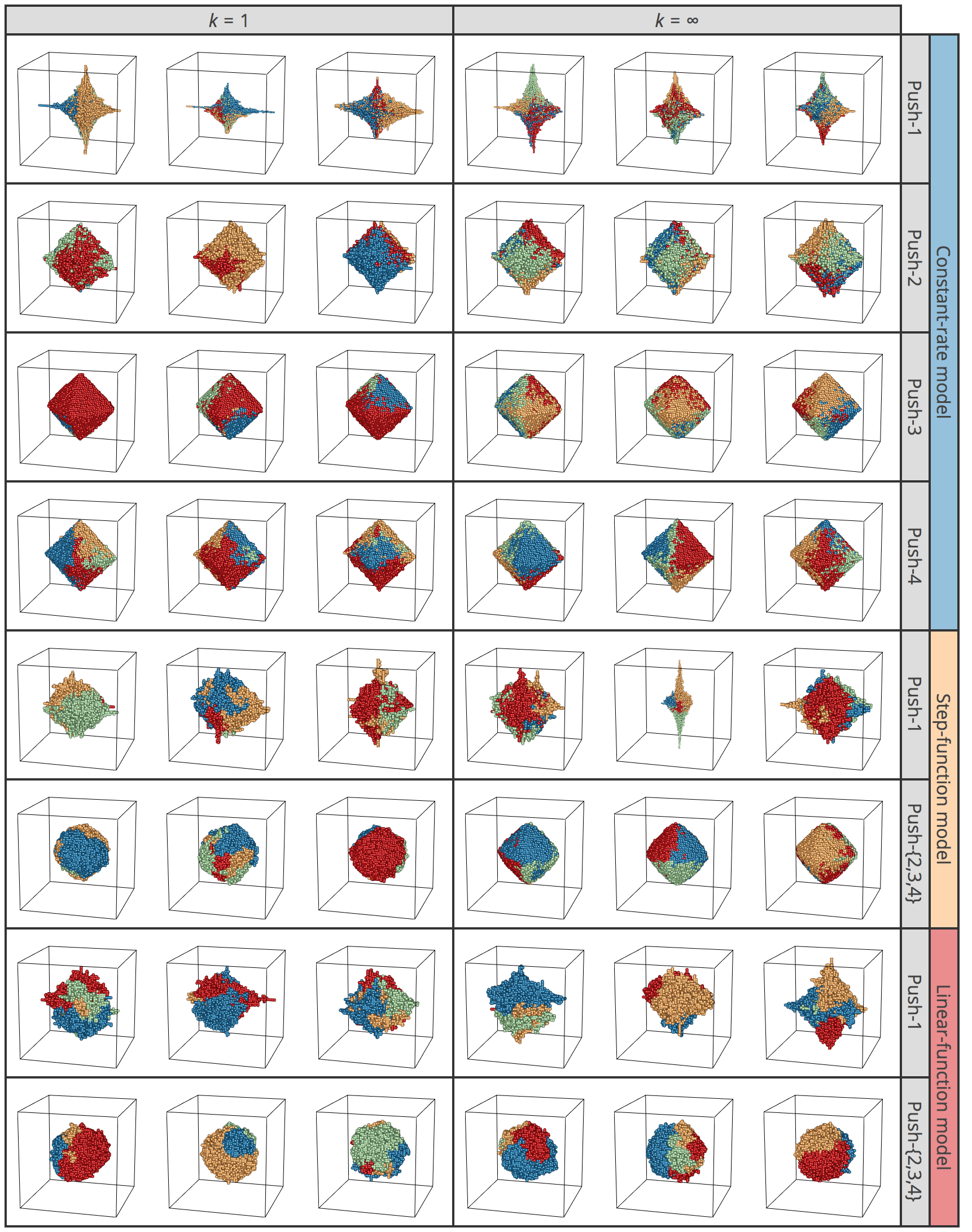

Supplement: S2 Fig — All parameters except for the lattice/neighborhood are the same as in Fig 8. (TIF) [file pone.0184229.s002.tif]

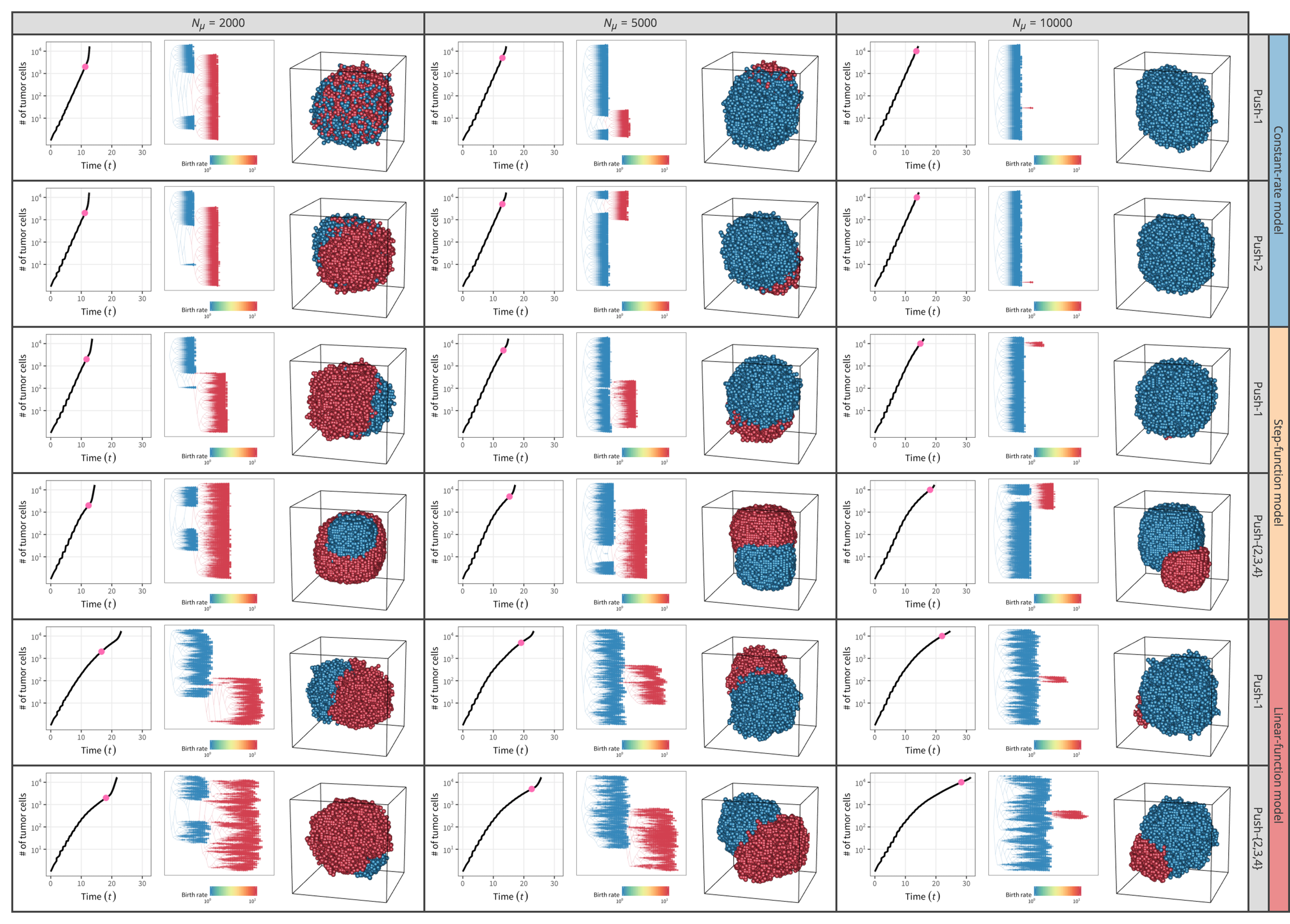

Supplement: S3 Fig — Results for push methods 1 and 2 under constant-rate, step-function, and linear-function models are shown; The cells with the string driver mutation (sβ = 9) are in red, while the others are in blue. All parameters except for sβ are the same as in Fig 13. (TIF) [file pone.0184229.s003.tif]
